# Searching for Sharp Drops in the Incidence of Pandemic A/H1N1 Influenza by Single Year of Age

**DOI:** 10.1371/journal.pone.0042328

**Published:** 2012-08-02

**Authors:** Jessica Hartman Jacobs, Brett Nicholas Archer, Michael G. Baker, Benjamin J. Cowling, Richard T. Heffernan, Geoff Mercer, Osvaldo Uez, Wanna Hanshaoworakul, Cécile Viboud, Joel Schwartz, Eric Tchetgen Tchetgen, Marc Lipsitch

**Affiliations:** 1 Department of Epidemiology, Harvard School of Public Health, Boston, Massachusetts, United States of America; 2 National Institute for Communicable Diseases, National Health Laboratory Service, Johannesburg, South Africa; 3 Department of Public Health, University of Otago, Wellington, New Zealand; 4 School of Public Health, The University of Hong Kong, Hong Kong Special Administrative Region, China; 5 Division of Public Health, Wisconsin Department of Health Services, Madison, Wisconsin, United States of America; 6 National Centre for Epidemiology and Population Health, Australian National University, Canberra, Australia; 7 National Institute of Epidemiology, ANLIS, Ministry of Public Health, Buenos Aires, Argentina; 8 Department of Disease Control, Ministry of Public Health, Tiwanond, Nonthaburi, Thailand; 9 Division of International Epidemiology and Population Studies, Fogarty International Center, National Institutes of Health, Bethesda, Maryland, United States of America; 10 Department of Environmental Health, Harvard School of Public Health, Boston, Massachusetts, United States of America; 11 Department of Biostatistics, Harvard School of Public Health, Boston, Massachusetts, United States of America; 12 Department of Immunology and Infectious Diseases, Harvard School of Public Health, Boston, Massachusetts, United States of America; University of British Columbia, Canada

## Abstract

**Background:**

During the 2009 H1N1 pandemic (pH1N1), morbidity and mortality sparing was observed among the elderly population; it was hypothesized that this age group benefited from immunity to pH1N1 due to cross-reactive antibodies generated from prior infection with antigenically similar influenza viruses. Evidence from serologic studies and genetic similarities between pH1N1 and historical influenza viruses suggest that the incidence of pH1N1 cases should drop markedly in age cohorts born prior to the disappearance of H1N1 in 1957, namely those at least 52–53 years old in 2009, but the precise range of ages affected has not been delineated.

**Methods and Findings:**

To test for any age-associated discontinuities in pH1N1 incidence, we aggregated laboratory-confirmed pH1N1 case data from 8 jurisdictions in 7 countries, stratified by single year of age, sex (when available), and hospitalization status. Using single year of age population denominators, we generated smoothed curves of the weighted risk ratio of pH1N1 incidence, and looked for sharp drops at varying age bandwidths, defined as a significantly negative second derivative. Analyses stratified by hospitalization status and sex were used to test alternative explanations for observed discontinuities. We found that the risk of laboratory-confirmed infection with pH1N1 declines with age, but that there was a statistically significant leveling off or increase in risk from about 45 to 50 years of age, after which a sharp drop in risk occurs until the late fifties. This trend was more pronounced in hospitalized cases and in women and was independent of the choice in smoothing parameters. The age range at which the decline in risk accelerates corresponds to the cohort born between 1951–1959 (hospitalized) and 1953–1960 (not hospitalized).

**Conclusions:**

The reduced incidence of pH1N1 disease in older individuals shows a detailed age-specific pattern consistent with protection conferred by exposure to influenza A/H1N1 viruses circulating before 1957.

## Introduction

Consistent with earlier pandemics of the 20^th^ century [1], [2], surveillance reports of hospitalized cases, laboratory confirmed cases, and mortality due to the first wave of novel 2009 pandemic influenza A/H1N1 (pH1N1) virus infection suggest a markedly younger age distribution than typically observed during seasonal influenza epidemics [3], [4], [5], [6]. During seasonal influenza epidemics, an estimated 90% of influenza-associated deaths occur among people aged >65 years [7]. In contrast, the global experience during the early months of the 2009 pandemic was a median age of 37 years in confirmed fatal cases (n = 343 cases) with the majority occurring in individuals aged 20–49 years [6]. Surveillance for hospitalized and laboratory confirmed pH1N1 cases also showed the inverse pattern of seasonal influenza, with the youngest age groups dominating incidence estimates and case counts. Only five percent of the first 272 patients hospitalized in the United States from pH1N1 were aged >65 years [4]. In a comparison of confirmed cases of pH1N1 from 10 countries on five continents the age distribution was consistent between countries and the largest source of variability was between continents [5]. About 75% of these cases occurred in persons aged <30 years with a small peak in ages 10–19 years; less than 3% of cases occurred in the elderly (≥65 years) [5].

The global surveillance data suggest that being an older adult is protective against pH1N1 infection and hospitalization. The risk of pH1N1-associated death among the elderly who were hospitalized was slightly elevated compared to younger age groups but the overall risk of death was much less so than in seasonal influenza [8]. The reduced risk of pH1N1-associated disease in the elderly population is likely the result of some level of immunity provided by cross-reactive antibodies generated from prior vaccination or infection with antigenically similar influenza A viruses [9]. Combined with genetic and antigenic studies demonstrating the similarities between pH1N1 and the descendants of the 1918 virus, the incidence of pH1N1 cases should drop markedly in adults born prior to versus after the disappearance of H1N1 in 1957, namely those at least 52–53 years old in 2009 [9], [10], [11].

To date, all published incidence data have used large age categories due to the small numbers of confirmed cases in each country or region. In order to evaluate whether sharp drops associated with the protective effects of earlier exposure do indeed exist, incidence should be compared across single-year age groups. To test for any age associated discontinuities in the incidence of laboratory-confirmed pH1N1 we analyzed data from 8 jurisdictions in 7 countries, stratified by single year of age, sex, and hospitalization status. We quantified sharp drops in incidence by looking for statistically significant negative second derivatives in the incidence risk with respect to age.

## Methods

### Data Sources

We obtained counts of laboratory confirmed cases of pH1N1 infection by single year of age and hospitalization status from Argentina, Australia (Queensland), Hong Kong, New Zealand, South Africa, Thailand, and the United States (Wisconsin and New York City). All locations used a real time reverse transcription polymerase chain reaction (RT-PCR) test to confirm cases of pH1N1. The data were collected as part of routine surveillance for pH1N1 conducted by the Ministries/Departments of Health in each location, and were reported to us anonymously as aggregated data covering many months (length of time varied by location). Since the investigators of this study had no interaction with patients and received no identifiable private information as part of this study, we were not required to obtain ethics approval or individual patient consent by the Harvard School of Public Health institutional review board under the United States Department of Health and Human Services’ regulations on human subjects. These cases were reported in the first complete wave of the pandemic for each location, under different testing protocols and levels of surveillance, and subject to differing biases, yet were analyzed together to have large cohorts within each age to identify discontinuities. In Wisconsin, RT-PCR confirmed influenza A cases that were not subtyped were included for the period June 1 to June 13, 2009, when testing confirmed that over 99.5% of subtyped viruses were pH1N1; these unsubtyped influenza A isolates were considered probable pH1N1 infections. In New York City, 67/996 (7%) of cases were designated as probable, defined as confirmed influenza A and negative for seasonal subtypes but lacking confirmatory pH1N1 testing [12]. Argentina, Hong Kong and Wisconsin further reported cases by sex. The case data used in this study from Argentina, Hong Kong, and Wisconsin are included as an online supplement (Table S1). The hospitalization status was unknown for South Africa and these data were not included in the weighted incidence risk ratios but are reported separately.

Collaborators in several locations additionally provided estimates of the 2009 population by single year of age. The populations of South Africa [13] and Thailand were available in 5 year age groups, so we applied the Sprague Multiplier to interpolate to population size for single year of age [14]. The 2010 census population of Argentina by single year of age and sex was obtained from the National Institute of Statistics and Censuses [15].

### Calculation of a Weighted Incidence Risk Ratio

We generated incidence risk ratios (RR) for each single year of age, hospitalization status, and location, dividing the incidence risk for each age group by the total for all age groups in that location and hospitalization status to normalize for differences in reporting. Thus the RR represents the risk of being a pH1N1 case for a person of a specific age relative to the overall risk in all ages combined. The variable sampling periods between locations and difficulty in defining person time at risk for an infectious disease where the true disease incidence is unknown required the use of cumulative incidence instead of an incidence rate calculation. The RR was defined as follows for each hospitalization status (*H* = *h* for 1 =  hospitalized cases and 2 =  not hospitalized), age (*I = i* from 0 to ≥100 years old) and location (*L = l* for the 7 locations exclusive of South Africa). The risk (*r_h|i,l_*) for each *h* given age  = *i* and location  = *l* was calculated using all confirmed cases (*x_i,h,l_*) of each age in a location and hospitalization status divided by the population for that age and location, as in equation (1). Similarly, an all age risk (*R_h|l_*) was calculated for each location and hospitalization status by summing the cases over all ages and dividing by the total population in that location. The RR for each age, hospitalization status and location (*RR_i,h|l_*) was then calculated by dividing (*r_h|i,l_*) by (*R_h|l_*), as demonstrated in equation (1).


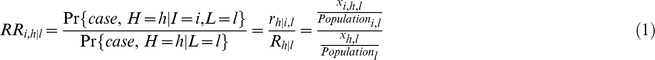


A weighted risk ratio (WRR) was then calculated for each age and hospitalization status where location specific RRs were weighted relative to their contribution to the total number of hospitalized or not hospitalized cases. The weights were comparable to an inverse variance weighting, where locations contributing higher case counts were more heavily weighted than those with smaller counts. The weights (*w_h,l_*) were calculated as follows in equation (2), using the previously described nomenclature:


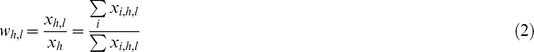


The final product was the weighted risk ratio (*WRR_i,h_*) for each age and hospitalization status, calculated as follows in equation (3):


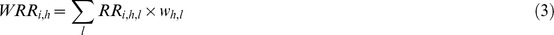


Since cases were stratified by sex in Argentina, Hong Kong, and Wisconsin, we also created *WRR_i,h_* by sex for these locations. Wisconsin had a small number of hospitalized cases and was only included in the *WRR_i_* calculation for cases that were not hospitalized. In addition, we compared the cumulative incidence of being a male versus female among <18, 18–64, and >64 year olds cases that were and were not hospitalized. These subanalyses allowed us to explore possible alternative mechanisms for any significant changes in incidence by age, including gender related exposure to pH1N1 or biological differences between the sexes in immunologic response to pH1N1.

### Graphical and Statistical Analysis of the Weighted Risk Ratios

To detect sharp drops in incidence by age, we searched for statistically significant, negative second derivatives in the smoothed WRR, with respect to time, reasoning that these would correspond to departures from underlying linear incidence trends with age. Using the SiZer package version 0.1–4.0 [16] in the statistical software R [17], we examined the first (1D) and second derivatives (2D) of the smoothed WRR as a function of age. SiZer is a tool for quantitatively identifying whether features of a data series rise above the level of noise. It is different from traditional approaches of smoothing and statistical inference because SiZer removes the bias inherent in selecting a bandwidth and allows an inspection at a wide range of smoothing bandwidths to see which features are insensitive to bandwidth selection and likely to be true features [18]. Small bandwidths can result in undersmoothing with large variances but low bias, as only the local data points are used to estimate the smoothed curve. In contrast, a large bandwidth oversmooths the data points and results in low variance but large bias, since many local data points are used which might not represent the local phenomenon. A true feature in a data series will persist across bandwidths.

The smoothing method employed in SiZer is a locally weighted polynomial regression (LWPR) using a Gaussian kernel at bandwidths (*b*) that vary per the user’s specifications. We allowed *b* to vary from 1.5 to 10 years and chose a second degree polynomial to be fit to the WRR*_i,h_* at each age. We specified that the values of the LWPR smoothed WRR*_i,h_* be evaluated at each integer of age (0–100 years). SiZer looks across a range of *b* and classifies the 1D and 2D as significantly positive, possibly zero or significantly negative. The choice of *b* determines how many neighbors are used to generate the LWPR. In our range of bandwidths, the effective sample size varies from 4 to 25 years. At *b* = 4, the effective sample size is 10 and is similar to smoothing over a decade of age. We also plotted the smoothed WRR*_i,h_* and the 1D and 2D using a fixed *b* of 1, 2, and 4 with a polynomial of degree 2.

## Results

### Hospitalized Cases

**Figure 1:**
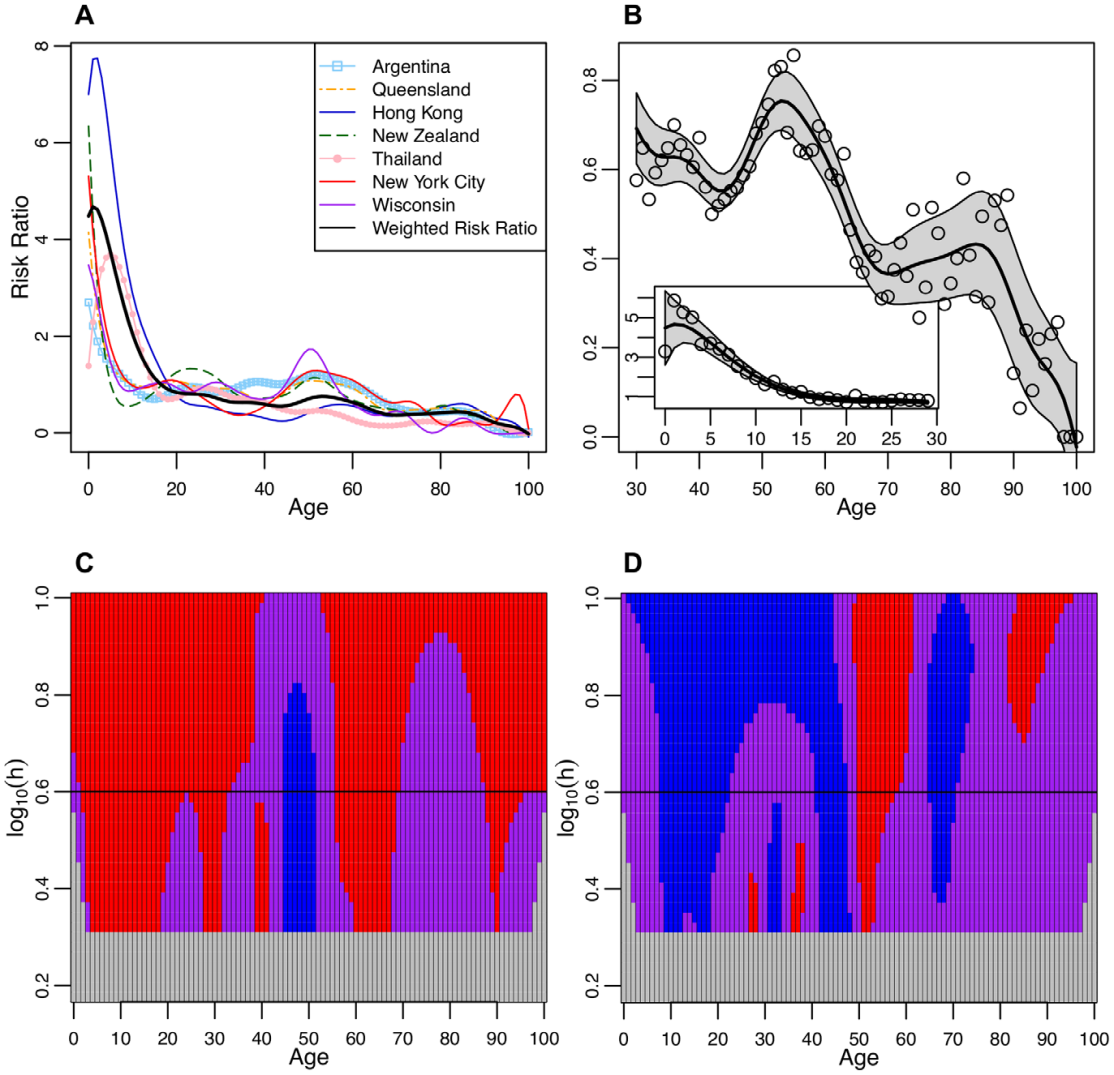
Laboratory confirmed hospitalized cases. **A** The smoothed risk ratio of laboratory confirmed hospitalized cases in a single year age group compared to the overall risk in all age groups. Smoothed curves for each location were created by a locally weighted polynomial regression with fixed bandwidth of 4. **B** The smoothed weighted risk ratio (WRR) of laboratory confirmed hospitalized cases in a single year compared to the risk in all age groups combined using a fixed bandwidth of 4. The single year of age WRR used to create the smoothed curve are plotted as open circles and the 95% confidence bounds are shaded. The inset figure shows the truncated WRR from 0 to 29 years of age while the larger figure focuses on the ages from 30–100. **C** SiZer plot of the first derivative of the WRR by age. The X axis represents age while the Y axis corresponds to the log of the bandwidth (h). For example, log(0.6) corresponds to the fixed bandwidth of 4 used to create Figures *A* and *B* and a black horizontal line identifies this bandwidth. The shading corresponds to the significance and direction of the slope (first derivative) of the WRR by age: red is significantly decreasing, purple is possibly zero, blue is significantly increasing, and light grey represents areas where there is insufficient data to generate a smoothed curve. The grid lines correspond to 1 year of age intervals. **D** SiZer plot of the second derivative of the WRR by age, where the shading corresponds to that described for Figure 1C.

Seven locations contributed surveillance records of hospitalized cases for a total of 18,788 pH1N1 hospitalizations (*Table 1*). Hong Kong, Thailand, and Argentina contributed 40, 24, and 22% of the hospitalized cases, respectively. The hospitalized cases had a similar age distribution to the previously published surveillance reports described above (*Table 1*). The RR was highest among children aged 2 years and younger in all locations but Thailand, where it peaked at age 5 (*Table 2* *and* *Figure 1A*). The magnitude of the age-specific relative risks between locations is not solely due to differences in disease burden but likely also reflects differences in surveillance (active versus passive), criteria for hospitalization, and changing protocols and recommendations as the pandemic wave progressed. Of greater interest than the absolute difference between locations for a given age is the risk relative to other ages within a place and whether the trend of RRs by age persists regardless of geography.

**Table 1:** Confirmed cases of 2009 pandemic A/H1N1 influenza by location and hospitalization status, the associated weights used to calculate the weighted risk ratio (shown as %), and the cumulative incidence risk ratio (95% confidence intervals) of male versus female cases by hospitalization status and age group for locations where sex was known.

	Hospitalized	Not Hospitalized	M:F Risk Ratio	M:F Risk Ratio
	(%)	(%)	Hospitalized^1^	Not Hospitalized^2^
**Location**				
Argentina	4,068 (21.7)	2,586 (6.0)		
Australia, Queensland	726 (3.9)	10,820 (24.9)		
Hong Kong	7,425 (39.5)	21,330 (49.1)		
New Zealand	991 (5.3)	2,202 (5.1)		
Thailand	4,421 (23.5)	2,169 (5.0)		
United States, NYC	996 (5.3)			
United States, Wisconsin	161 (0.9)	4,319 (9.9)		
**Age Group in years**				
<18	9,794 (52.1)	22,618 (52.1)	1.16 (1.10, 1.22)	1.24 (1.11, 1.38)
18–64	8,175 (43.5)	20.503 (47.2)	0.86 (0.81, 0.91)	0.85 (0.82, 0.88)
>64	819 (4.4)	305 (0.7)	1.72 (1.47, 2.03)	0.52 (0.37, 0.72)
**Total**	18,788	43,426		

1 An unweighted cumulative incidence risk ratio determined using lab confirmed hospitalized cases from Argentina and Hong Kong.

2 An unweighted cumulative incidence risk ratio determined using lab confirmed cases that were not hospitalized from Argentina, Hong Kong, and Wisconsin.

**Table 2:** Peak risk ratio by location, age and hospitalization status.^1^

	Hospitalized Peak RR (Age)	Not Hospitalized Peak RR (Age)
**Location**		
Argentina	3.3 (<1)	2.0 (6)
Australia, Queensland	4.6 (<1)	2.2 (7)
Hong Kong	11.1 (2)	4.2 (6)
New Zealand	7.1 (<1)	2.8 (19)
Thailand	4.4 (5)	3.3 (6)
United States, NYC	6.2 (<1)	
United States, Wisconsin	3.9 (2)	3.8 (9)
Overall Weighted Risk Ratio	5.9 (1)	3.2 (7)

1 This is the actual risk ratio, not the peak in the locally weighted polynomial regression smoothed risk ratio.

The decline in risk from infancy towards adulthood plateaus around the early thirties and the risk begins to increase from 45 years of age to the early fifties (*Figures 1B and 1C*). This increase in the overall trend is consistent through all locations – with an additional relative maximum in the early to mid twenties occurring in several locations as well. The risk peaks again at 53 years of age and then begins to decline rapidly, with the rate of decline accelerating until 60 years of age (*Figure 1D*). These features persist throughout varying bandwidths, whether the effective sample size of neighbors included is 5.25 years (*b* = 2.1, the lowest bandwidth where there is enough data to create a smoothed curve) or 25 years (*b = *10, the highest bandwidth we explored on the SiZer plot). The WRR smoothed with a bandwidth of 4 gave the visually optimal fit in terms of capturing most data points while removing the less interesting noise. The second derivative of the smoothed WRR suggests that there is a statistically significant drop in the slope of the WRR (in fact, switching from positive slope to negative slope) in individuals between the ages of 50 to 58 years (born between 1951 and 1959).

### Cases that were not Hospitalized

**Figure 2:**
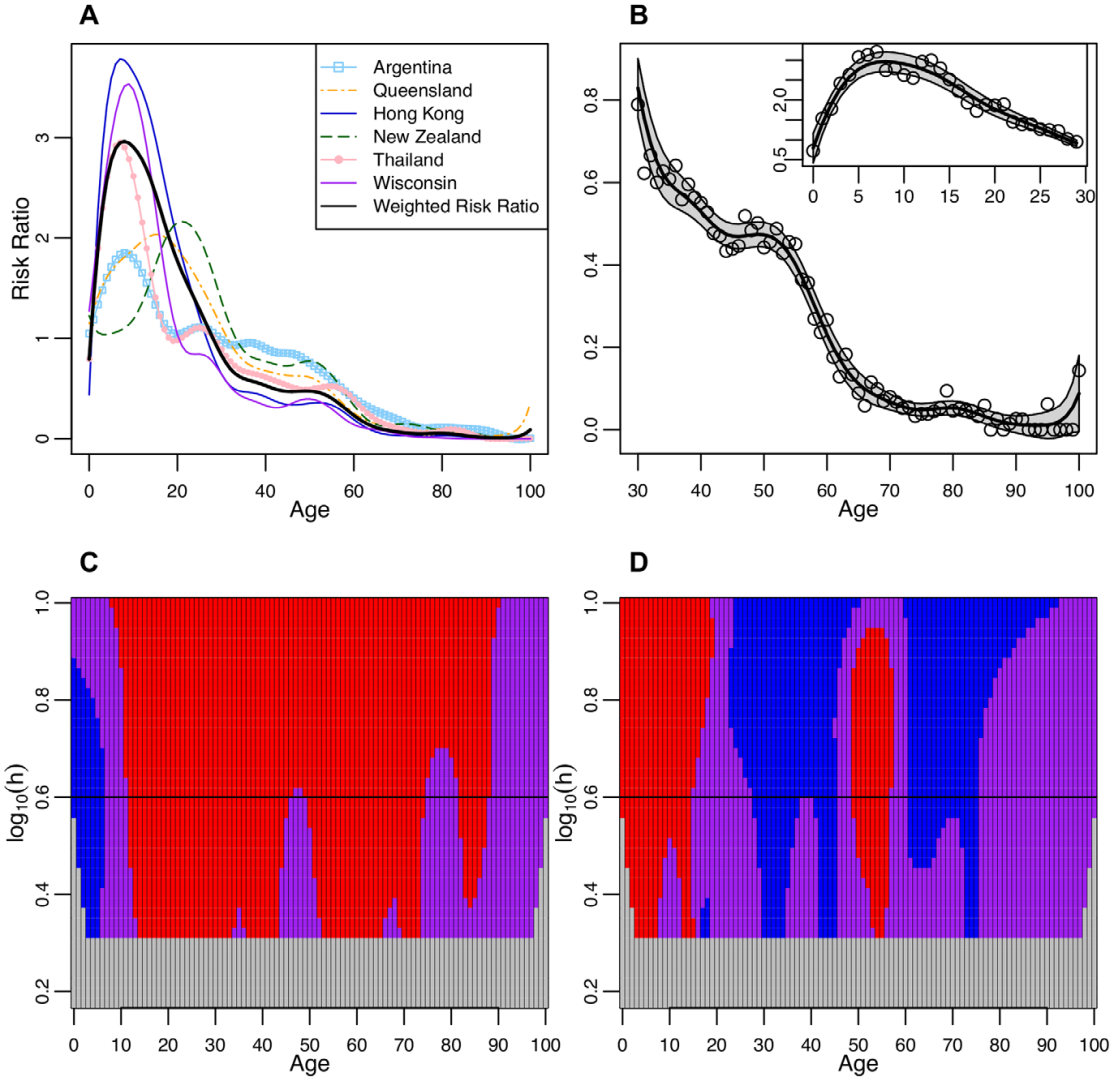
Laboratory confirmed cases that were not hospitalized. **A** The smoothed risk ratio of laboratory confirmed cases that were not hospitalized in a single year age group compared to the overall risk in all age groups. Smoothed curves for each location were created by a locally weighted polynomial regression with fixed bandwidth of 4. **B** The smoothed weighted risk ratio of cases that were not hospitalized in a single year compared to the risk in all age groups combined using a fixed bandwidth of 4. The single year of age weighted risk ratios used to create the smoothed curve are plotted as open circles and the 95% confidence bounds are shaded. The inset figure shows the truncated WRR from 0 to 29 years of age while the larger figure focuses on the ages from 30–100. **C** SiZer plot of the first derivative of the weighted risk ratio by age. The X axis represents age while the Y axis corresponds to the log of the bandwidth (h). For example, log(0.6) corresponds to the fixed bandwidth of 4 used to create Figures *A* and *B* and a black horizontal line identifies this bandwidth. The shading corresponds to the significance and direction of the slope (first derivative) of the weighted risk ratio by age: red is significantly decreasing, purple is possibly zero, blue is significantly increasing, and light grey represents areas where there is insufficient data to generate a smoothed curve. The grid lines correspond to 1 year of age intervals. **D** SiZer plot of the second derivative of the weighted risk ratio by age, where the symbols are as described for Figure *2C*.

Six locations contributed to the count of confirmed pH1N1 cases that were not hospitalized for a total of 43,426. Hong Kong and Queensland, Australia contributed almost 75% of these infections (*Table 1*). The distribution of cases between the three broad age groups (<18, 18–64, and 65 and older) looks very similar between cases that were and were not hospitalized, with 52.1% of the cases being in the youngest age group. Only a very small percentage of the cases that did not require hospitalization were older than 64 years (0.7%). In contrast to the hospitalized cases, the peak in risk amongst the differing locations is shifted to slightly older children and appears between 6 and 19 years of age (*Table 2* *and* *Figure 2A*). The range in peak RRs among locations is narrower in cases that were not hospitalized (2.0–4.2).

**Figure 3:**
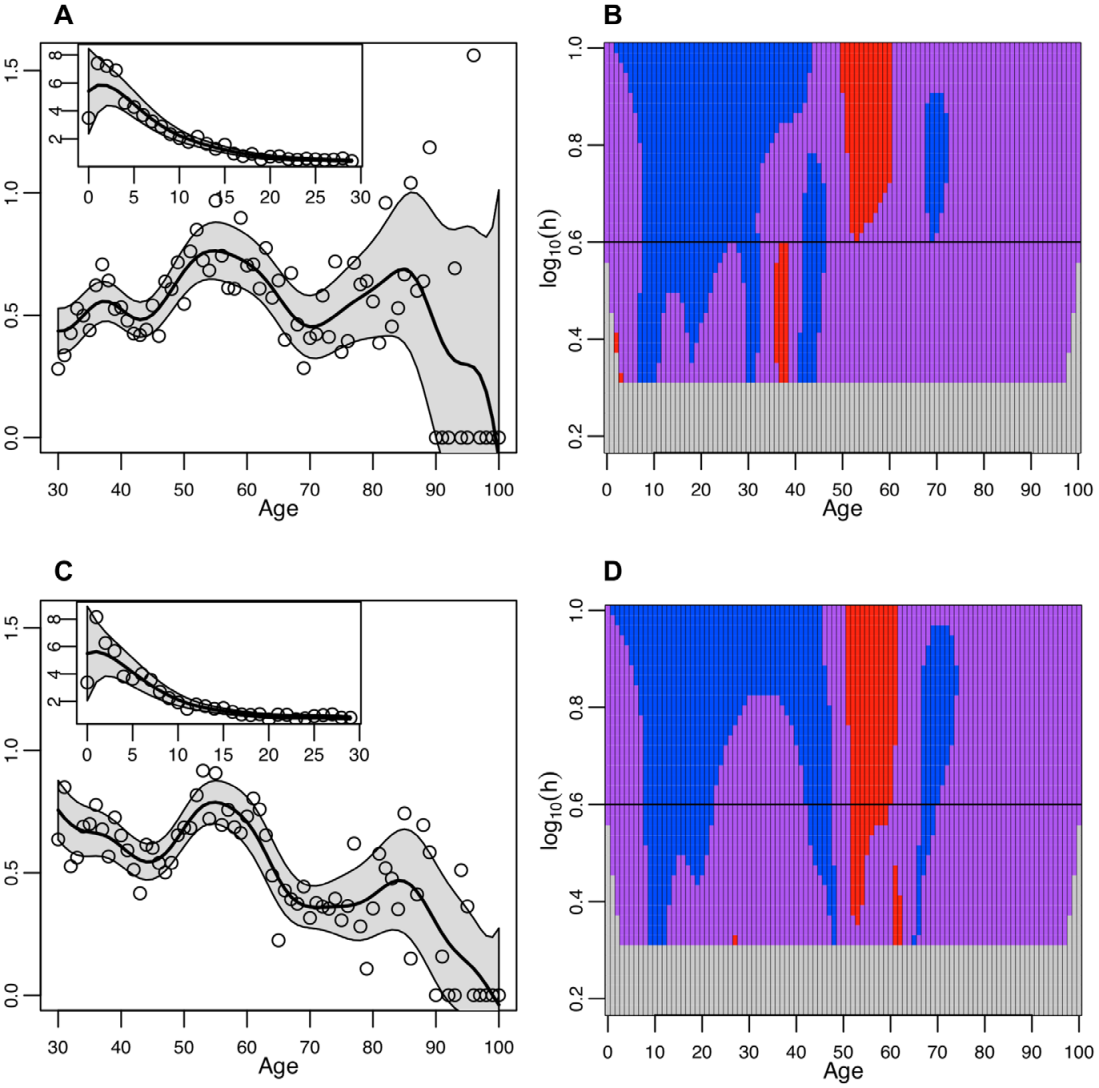
Differences by sex in hospitalized cases. **A Hospitalized Men.** The smoothed risk ratio of laboratory confirmed hospitalized cases among men in a single year age group compared to the overall risk in all male age groups. Smoothed curves were created by a locally weighted polynomial regression with fixed bandwidth of 4. The single year of age weighted risk ratios used to create the smoothed curve are plotted as open circles and the 95% confidence bounds are shaded. The inset figure shows the truncated WRR from 0 to 29 years of age while the larger figure focuses on the ages from 30–100. **B Hospitalized Men.** SiZer plot of the second derivative of the weighted risk ratio by age among male hospitalized cases. **C Hospitalized Women.** The smoothed risk ratio of laboratory confirmed hospitalized cases among women in a single year of age compared to the overall risk in all female age groups. Smoothed curves were created by a locally weighted polynomial regression with fixed bandwidth of 4. The single year of age weighted risk ratios used to create the smoothed curve are plotted as open circles and the 95% confidence bounds are shaded. The inset figure shows the truncated WRR from 0 to 29 years of age while the larger figure focuses on the ages from 30–100. **D Hospitalized Women.** SiZer plot of the second derivative of the weighted risk ratio by age among female hospitalized cases.

The WRR of cases who were not hospitalized peaks in 8 year olds and then declines nearly continuously until the early eighties (*Figure 2B and 2C*). The WRR flattens out briefly around 47–48 years of age but then declines again until 75 years of age where the slope is zero until 82 years when the WRR declines further. The rate of decline of the WRR slows dramatically from 28 to 45 years of age but then this trend reverses between 49 and 56 years and the rate of decline in the WRR accelerates (*Figure 3D*). From 61 to 75 years the rate of decline again slows down and becomes stable after 75 years of age. The WRR suggests that incidence begins to stabilize among adults in their forties, but that around 49 years of age the incidence declined rapidly.

In South Africa (N = 12,497), the age distribution of confirmed cases (in which hospitalization status was not recorded) looks similar to the distribution of cases that were not hospitalized, suggesting that the majority of reports had not been hospitalized (data not shown). The patterns observed in South Africa also mirror those of cases that were not hospitalized (Figure S1). A peak in risk occurs at 14 years of age (RR = 4.2) and then declines until 31 years, with the rate of decline significantly decreasing between 21 and 35 years of age (Figure S1). The RR plateaus between 32–45 years of age but then a rapid acceleration in the rate of decline occurs between 41–52 years of age at slightly higher bandwidths. The RR declines steadily from 46–73 years of age and then it plateaus again.

### Role of Sex

To explore potential sex variation in age-specific incidence risk, we further stratified the datasets from Argentina, Hong Kong, and Wisconsin, for which sex information was available. Among men hospitalized for pH1N1-related disease, the WRR declines from 4 to 28 years of age but then stabilizes and begins to rise again from 44 to 52 years of age before reaching a plateau again (*Figure 3A*). A final decline in the WRR occurs from 61 to 67 years of age but the WRR is then stable until 100 years of age. There is a narrow acceleration in the rate of decline around 53 years of age, but it is only statistically significant at larger bandwidths (*Figure 3B*). Among hospitalized women, a similar rise in WRR occurs between 46–52 years of age (*Figure 3C*), but then there is a rapid acceleration in the rate of decline between 52 and 60 year olds that is absent in men (*Figure 3D*).

**Figure 4:**
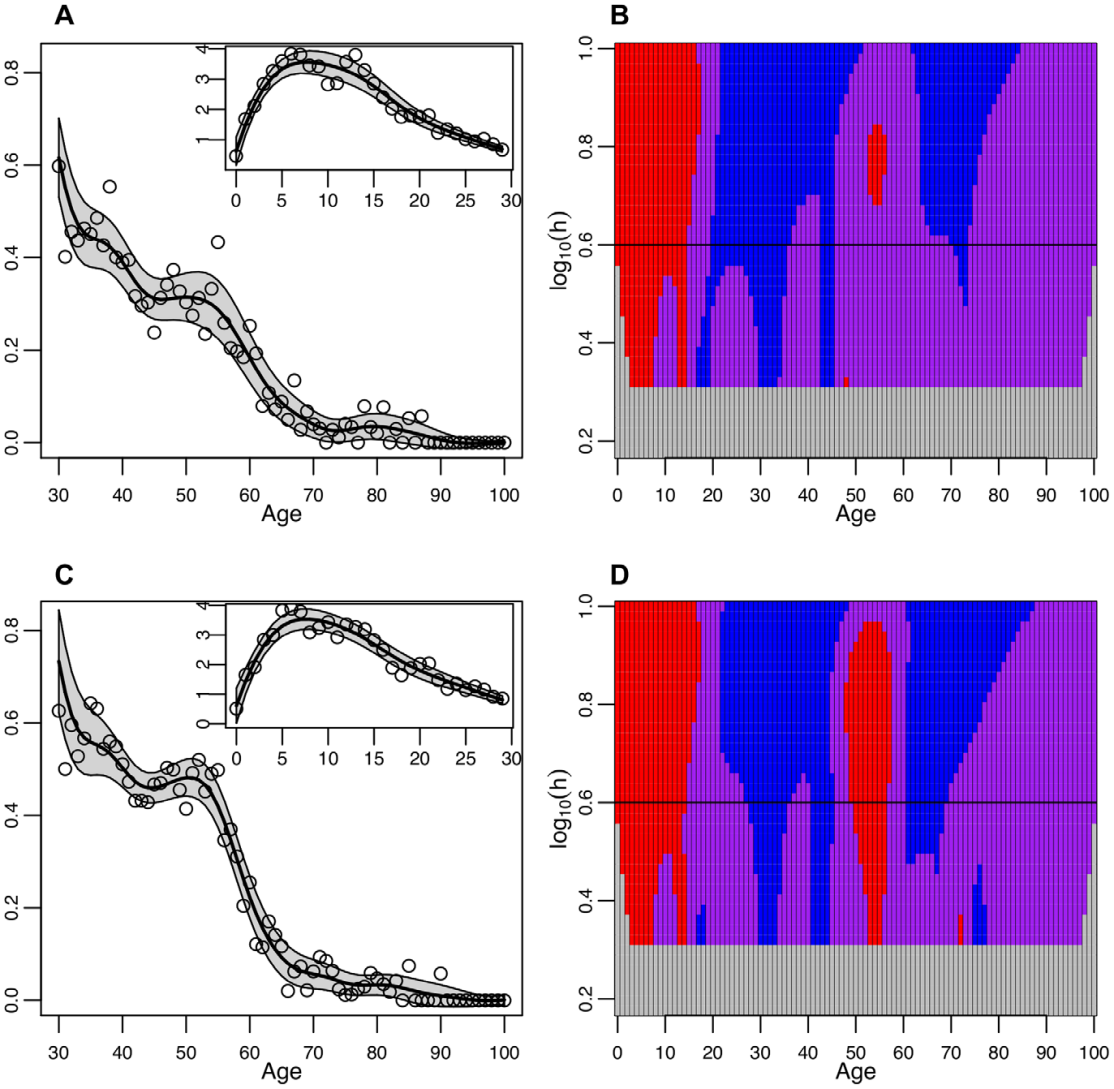
Differences by sex in cases that were not hospitalized. **A Men not hospitalized.** The smoothed risk ratio of cases among men who were not hospitalized in a single year age group compared to the overall risk in all age groups. Smoothed curves were created by a locally weighted polynomial regression with fixed bandwidth of 4. The single year of age weighted risk ratios used to create the smoothed curve are plotted as open circles and the 95% confidence bounds are shaded. The inset figure shows the truncated WRR from 0 to 29 years of age while the larger figure focuses on the ages from 30–100. **B Men not hospitalized.** SiZer plot of the second derivative of the weighted risk ratio by age among men who were not hospitalized. **C Women not hospitalized.** The smoothed risk ratio of cases among women who were not hospitalized in a single year of age compared to the overall risk in all female age groups. Smoothed curves were created by a locally weighted polynomial regression with fixed bandwidth of 4. The inset figure shows the truncated WRR from 0 to 29 years of age while the larger figure focuses on the ages from 30–100. The single year of age weighted risk ratios used to create the smoothed curve are plotted as open circles and the 95% confidence bounds are shaded. **D Women not hospitalized.** SiZer plot of the second derivative of the weighted risk ratio by age among women who were not hospitalized.

This pattern of acceleration in the rate of decline among women (which is less pronounced among men) beginning in the early fifties and continuing until the early sixties occurs in cases that were not hospitalized as well. Among men who where not hospitalized the WRR increases initially and then declines from 11 to 45 years of age when the WRR starts to stabilize and then declines again from 54 to 72 years of age (*Figure 4A*). Among adult men, there is little statistical evidence of an acceleration in the rate of decline (*Figure 4B*). The WRR among women who were not hospitalized is similar to men who were not hospitalized (*Figure 4C*) with the exception that the rate of decline accelerates in a statistically significant way among women between the ages of 49–56 (*Figure 4D*).

Regardless of hospitalization status, male children had a higher risk of laboratory confirmed pH1N1 than females; the cumulative incidence risk ratio (95% confidence interval) of male versus females for hospitalized and not hospitalized cases was 1.16 (1.10, 1.22) and 1.24 (1.11, 1.38), respectively (*Table 1*). This pattern reversed in adults aged 18–64 years and women had a higher incidence of pH1N1 disease than men; 0.86 (0.81, 0.91) and 0.85 (0.82, 0.88) for hospitalized and not hospitalized. Among the elderly, men had an increased risk of hospitalization versus women while the opposite was true among cases that were not hospitalized; 1.72 (1.47, 2.03) and 0.52 (0.37, 0.72), respectively.

## Discussion

We have found evidence that the risk of laboratory confirmed pH1N1 infection declines with age, but that there is a statistically significant leveling off or increase in risk from about 45 to 50 years of age, after which a sharp drop in risk occurs until the late fifties. This trend was more pronounced in hospitalized cases and women, regardless of location. The age range at which the decline in risk accelerates corresponds to the cohort born between 1951–1959 (hospitalized) and 1953–1960 (not hospitalized). Although this is the first study describing the age patterns of pH1N1 cases and hospitalization by single year of age, our results are in broad agreement with previous studies [5], [19]. Several mechanisms, which are not mutually exclusive, could account for the rapid decline in influenza risk past 50 years of age: variation in prior immunity from earlier life exposure (cellular immunity and cross-reactive antibodies to conserved epitopes), in exposure to pH1N1 during the pandemic, and immune function related to aging and sex. We further discuss each in light of our findings.

The history of a person’s exposure to influenza A viruses determines their response to a new infection. It is not currently evident whether the clinical protection against pH1N1 observed among the elderly comes from prior immunity associated with their first encounter with an influenza virus (original antigenic sin) or from an accumulation of exposures to conserved epitopes in seasonal and older antigenically similar H1N1 that elicit a cellular and humoral immune response [11]. Clinical protection could result from both antibody-based protection from infection, which is determined by exposure to antigenically similar hemagglutinin (HA), and lessened disease severity, which is influenced by T cells and antibodies primed by multiple epitopes in the H1N1 virus. We do not have the capability to distinguish between these types of immune protection among the older adults in our study.

The theory of original antigenic sin stipulates that the first encounter with an influenza A virus in childhood sets the immune response to all other influenza A viruses in the future, which could explain the observed clinical protection against 2009 pH1N1 in seniors [20], [21]. Even when a person is exposed to an influenza virus that is antigenically dissimilar to the first virus they encountered, their immune system will mount a strong immune response to that first virus. If the theory of original antigenic sin holds, then anyone born prior to 1957 (especially those born several years prior, having lived through several H1N1 influenza seasons) would likely have had their first influenza A encounter with an H1N1 influenza virus and thus have some cross-protection to pH1N1. At a population level, this protection would increase past age 52 as the probability of a first infection with an H1N1 virus increases. In contrast, when H1N1 reemerged in 1977, it co-circulated with H3N2 viruses, which would have resulted in fewer individuals whose first exposure was to H1N1. This is consistent with the results of our study, where we saw no sharp decline in risk in the age cohort that was born after 1977 (those who were 32 in 2009) but a sharp decline in risk in those born prior to 1957.

If protection relied solely on exposure to a specific virus that has antigenic similarities to pH1N1, then we would have expected a sharp drop in risk in those that were born before 1943, or in adults 66 and older. The likely origin of the HA of the 2009 pH1N1 is a classical swine virus that has been relatively antigenically stable since it entered into swine around 1918 [22], [23]. The human 1918 and 2009 pH1N1 HA have the same neutralizing epitopes on the receptor-binding domain and both lack glycosylation sites in this region [24]. As the 1918 strain drifted in humans, glycosylation sites were added to the HA head and the current seasonal H1N1 strain has 2 glycosylation sites, so that there is no cross-reactivity between modern seasonal H1N1 strains and pH1N1 [25]. In contrast, the pH1N1 virus is antigenically most similar to human H1N1 viruses that circulated from around 1918 to the early 1940 s and classical swine H1N1 viruses [26]. Thus our study results suggesting a sharp decline in risk beginning at age 52, not age 66, suggests that immunologic protection is derived primarily from first exposure to any H1N1 virus and depends less on the antigenic similarity of the H1N1 strains.

Studies of pre-pandemic stored serum have provided fairly consistent evidence that cross-protective immunity from antibodies against pH1N1 increases with age, with the highest levels occurring in adults >60 years old [27], [28]. Comparability of serology studies published to date is hampered by a multitude of factors. These studies show a broad range in the age-specific prevalence of immune protection from prior H1N1 infection [9], [27], [29], [30], [31], [32]. Cross-protective immunity is most commonly defined as a hemagglutinin inhibition (HI) titer of >1∶40 or a microneutralization (MN) assay titer of >1∶160, which translates into a 50% reduction in influenza infection or disease in a population [27]. In the locations included in our study where pre-pandemic serology studies have been conducted, most conclude that around 20–30% of the population over 60 years old had pre-existing antibodies. In New Zealand, 22.6% (95% CI: 15.3–30%) of adults >60 years old (N = 124 samples) had pre-existing protection (determined by HI assay) [29]. In North Queensland, Australia, 19% (95% CI: 4–34%) of adults >65 years old (n = 27) had pre-existing immunity (determined by HI assay) while a larger study of 259 adults >60 years old in Australia found pre-existing immunity in 37.5% (95% CI: 31.6–43.3%) [33], [34]. In the United States, 34% of adults born before 1950 (N = 115) had cross-reactive antibodies to pH1N1 (determined by MN) [9] while in Hong Kong 37% (n = 30) adults >65 years showed seroprotective levels of antibodies to pH1N1 [35]. In Thailand, of 100 stored serum samples from persons aged 11–86 years, only 2 (both from adults aged >50 years), showed seroprotective HI assays, however it is unclear from the study how many adults >50 years old were sampled [36]. No serology studies have been published in Argentina.

In studies grouped by 10-year increments of age, there was evidence of cross-reactive antibodies in those born in the 1950 s, suggesting some circulation of H1N1 viruses that were antigenically similar to pH1N1. However, a positive cross-protective antibody serum test is only part of the immune response that has spared the elderly in this pandemic. If 20–30% of the population aged >50 years had HI assay titers >1∶40 and this corresponds to a 50% reduction in infection, we could expect a risk reduction for only 10–15% of this population. This reduction of risk is much less than that observed among the elderly during the pandemic and less than the risk reduction observed in our study.

While the HI and MN assays are good indicators of the immune response to an influenza virus, other antibody responses and the avidity of the antibodies produced also contribute to viral clearance from a host [37]. In one study, the elderly had memory B cells from prior exposure to 1918-like H1N1 viruses that were rapidly recruited, underwent selection, and affinity maturation when presented with pH1N1 vaccine, resulting in a quantitatively and qualitatively superior response than adults aged 18–65 years [37]. Given restrictions on the number of samples included in that study, the group of most interest for comparison with our study was aggregated into 46–64 years of age, which precludes a direct comparison with our study results.

Memory B cells isolated from survivors of the 1918 pandemic are capable of producing neutralizing antibodies against 1918 H1N1 and 1930 influenza A/Swine/Iowa/15/30, and to a lesser degree 1943 and 1977 H1N1 viruses, after surviving more than 90 years in the human body; this suggests that immunity to antigenically similar influenza A viruses is life long [38].

In addition to benefiting from immunologic protection resulting from prior H1N1 exposure, adults in their late fifties during the 2009 pandemic also likely benefitted from a lower exposure to school aged children; the age group with the highest attack rates [39]. We could not assess this possibility further as we did not have data on the number of children in the household for individual cases. However, despite different age and family structures, we did not find meaningful differences in the WRR between countries. This suggests that exposure to school aged children is not a significant determinant in the age-associated decline in incidence. An additional mechanism for the acceleration in WRR decline that we observed could be changes in the sex specific hormones that are dramatically altered in post-menopausal women. We explored the differences in WRR decline between men and women and found that regardless of hospitalization status, women had a statistically significant acceleration in decline of the WRR between 52–60 years (hospitalized) or 49–56 (not hospitalized), which was more pronounced than in men. The paucity of data on the response of post-menopausal women to influenza and in particular the role of sex hormones in the modulation of disease severity or susceptibility complicates our interpretation [40]. We cannot rule out a possible role of menopause in causing the sharp decline in risk that we observed in women more strongly than men in their fifties. Whether this effect is a main effect of menopause, or a modifying effect of menopause on the strength of acquired immunity cannot be assessed in this study.

The primary limitation in our study is that we have no information about the cases other than their age and incomplete data on sex. We do not know what their prior exposure to influenza A viruses (including wild- or vaccine-type exposures) has been, nor do we have information about comorbidities, family, and social structure. As such, we have no sense of the unique immunological history of each individual case. The immune response to influenza A viruses is complex and not well understood and operates in the landscape of other systems in a human body – i.e. two equally aged individuals with the exact same exposure history to influenza could have different responses to pH1N1 exposure based on other risk factors. Another limitation of our study is the lack of information on testing practices and healthcare seeking behaviors, and it is possible that there were age biases in propensities to test or in ability to detect influenza given infection, as well as gender biases in referral. Ideally, the age distribution of laboratory confirmed pH1N1 cases could be compared to that of seasonal influenza to adjust for such biases, but unfortunately no highly detailed age-specific dataset was available from earlier influenza seasons. Despite this complexity and the stratification of our study by only single year of age, the acceleration in the decline of incidence in cohorts born prior to 1957, consistently found in 8 international locations, is striking.

Our study has demonstrated that the relative risk of being a laboratory confirmed pH1N1 case levels off among adults aged 30 to late 40 and even increases among hospitalized cases, and then declines rapidly among adults in their fifties. Our results do not show an exact drop in those born before 1957 (i.e. 52 years of age in 2009) for several reasons. First, birth year is only an indication of exposure to H1N1 or prior infection with influenza H1N1 viruses; not everyone is exposed to influenza every year and the effect of these mechanisms should be spread out. Second, the use of a smoothing bandwidth of 4 could account for the smoothed WRR for cohorts born the three years after 1957 being involved in the observed rapid decline, as smoothing borrows information from the neighboring ages before and after the age for which it is estimating the WRR. Overall, our multinational dataset is most consistent with immune protection in people older than 52 years in 2009, resulting from priming with any A/H1N1 virus circulating before 1957, consistent with the theory of original antigenic sin. In addition, our data highlight gender variation in influenza risk by age that could be linked with changes in immune function due to menopause. Interestingly, these variations are not expected to be unique to the 2009 pandemic and hence the importance of menopause could be confirmed with data from seasonal outbreaks. Further experimental and epidemiological studies should shed light on the role of sex in the risk of influenza morbidity and mortality – a relatively new field of research [40].

## Supporting Information

Figure S1 **Confirmed Cases in South Africa.** **A** The smoothed weighted risk ratio (WRR) of laboratory confirmed cases in a single year compared to the risk in all age groups combined using a fixed bandwidth of 4. The single year of age WRR used to create the smoothed curve are plotted as open circles and the 95% confidence bounds are shaded. The inset figure shows the truncated WRR from 0 to 29 years of age while the larger figure focuses on the ages from 30–80+, where 5 cases in 80–90 year olds were aggregated into one single year of age. **B** SiZer plot of the first derivative of the WRR by age. The X axis represents age while the Y axis corresponds to the log of the bandwidth. For example, log(0.6) corresponds to the fixed bandwidth of 4 used to create Figures *A* and a black horizontal line identifies this bandwidth. The shading corresponds to the significance and direction of the slope (first derivative) of the WRR by age: red is significantly decreasing, purple is possibly zero, blue is significantly increasing, and light grey represents areas where there is insufficient data to generate a smoothed curve. The grid lines correspond to 1 year of age intervals. **C** SiZer plot of the second derivative of the WRR by age, where the shading corresponds to that described for Figure 1B.
(DOC)

Table S1 This table includes the cumulative cases for each single year of age (0–>99 years) by sex for three of the locations in this study. Included are Wisconsin, Hong Kong, and Argentina. The population is also included to allow the replication of these analyses.
(XLSX)
