# Supplementary figures and images for: Searching for Sharp Drops in the Incidence of Pandemic A/H1N1 Influenza by Single Year of Age
Source: PLoS One. 2012 Aug 2;7(8):e42328. doi: 10.1371/journal.pone.0042328 (PMC3410923; doi:10.1371/journal.pone.0042328)

**Figure S1. Confirmed Cases in South Africa.**


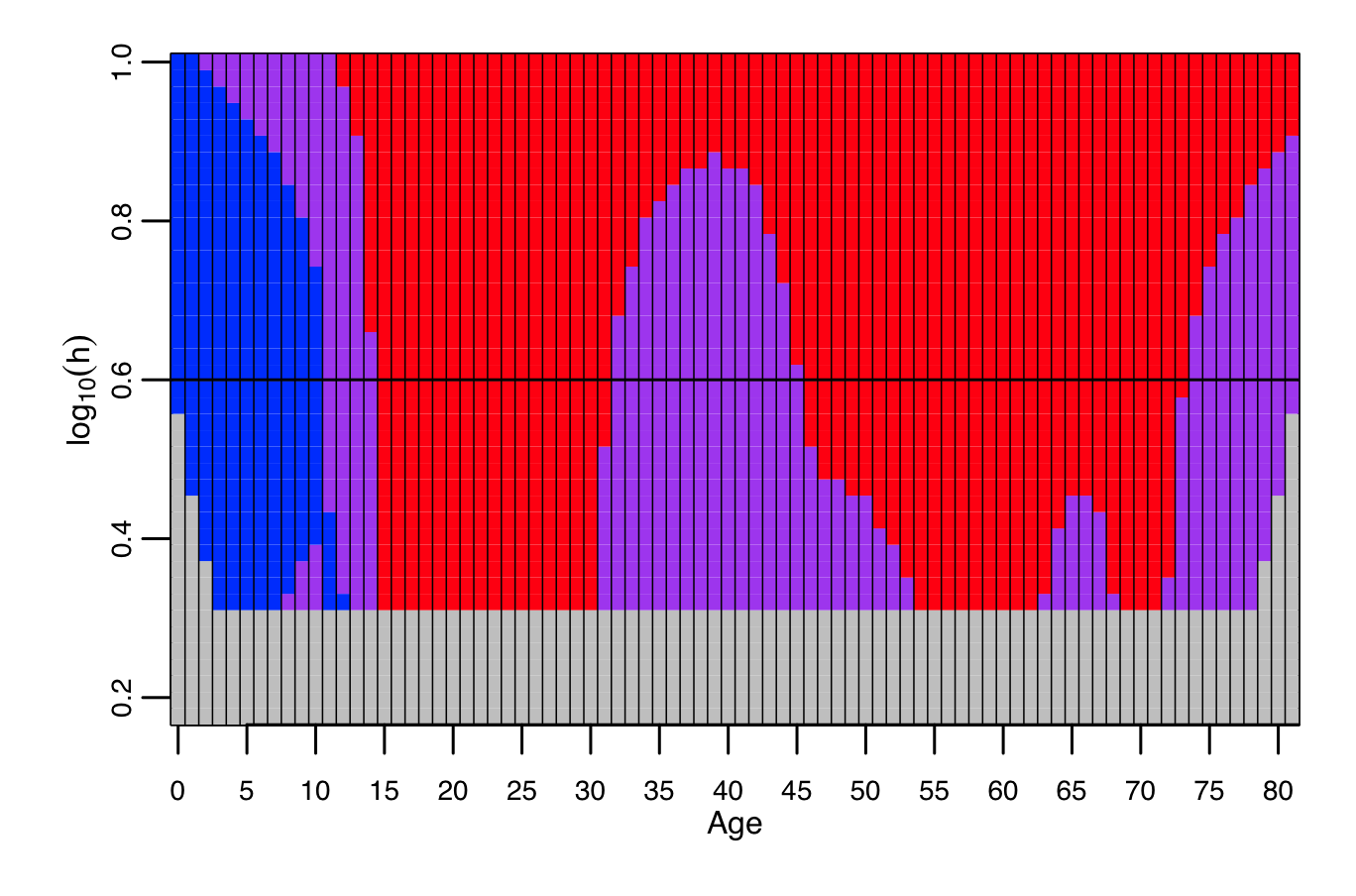

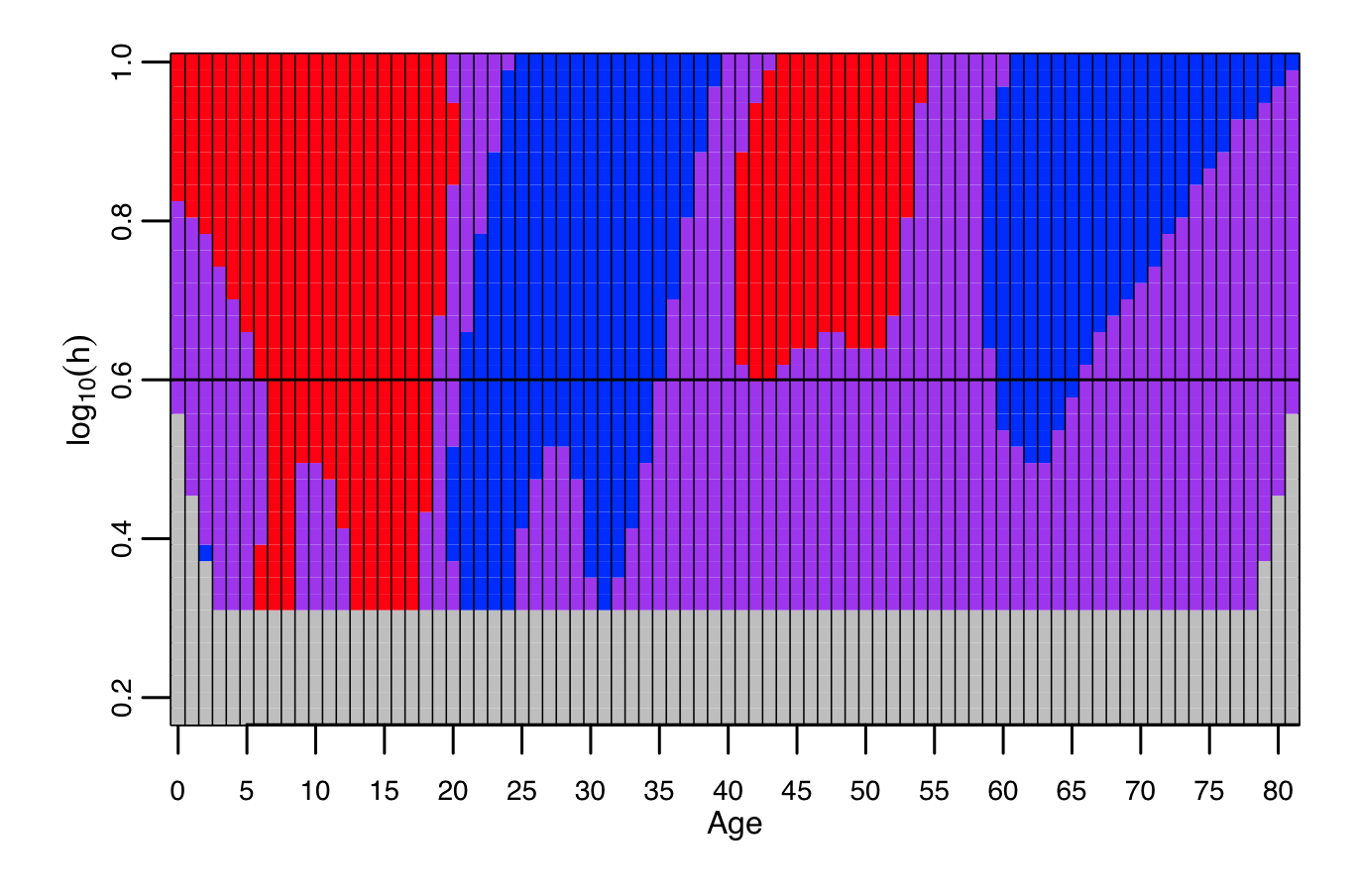
**A** **B**


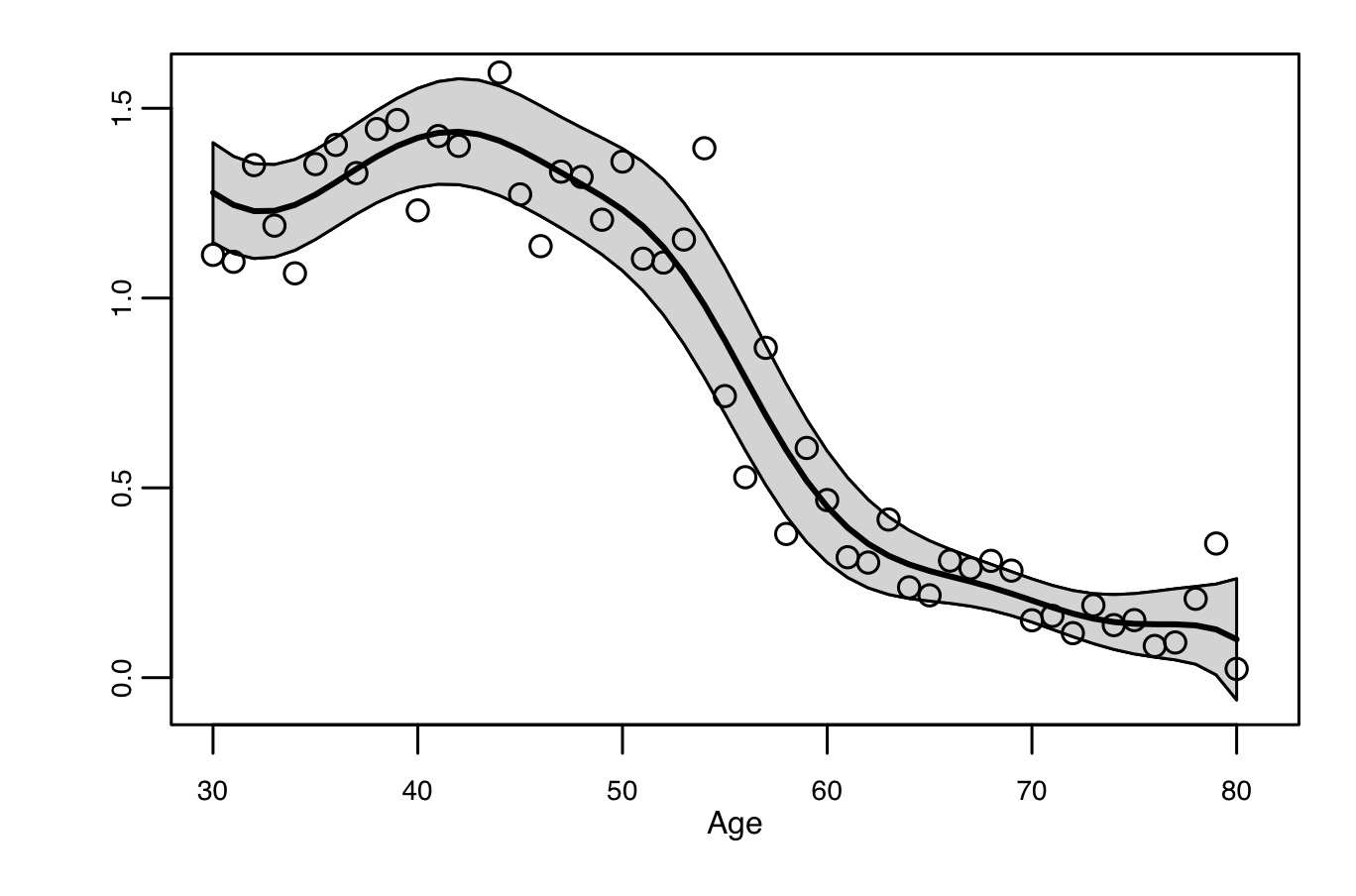

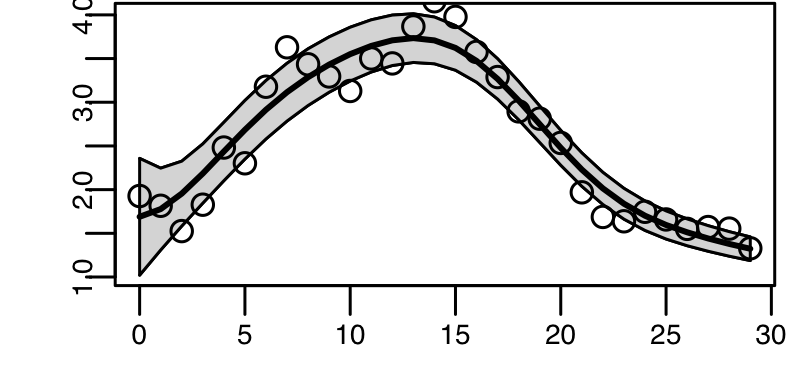


**C**

Supplement: Figure S1 — Confirmed Cases in South Africa. A The smoothed weighted risk ratio (WRR) of laboratory confirmed cases in a single year compared to the risk in all age groups combined using a fixed bandwidth of 4. The single year of age WRR used to create the smoothed curve are plotted as open circles and the 95% confidence bounds are shaded. The inset figure shows the truncated WRR from 0 to 29 years of age while the larger figure focuses on the ages from 30–80+, where 5 cases in 80–90 year olds were aggregated into one single year of age. B SiZer plot of the first derivative of the WRR by age. The X axis represents age while the Y axis corresponds to the log of the bandwidth. For example, log(0.6) corresponds to the fixed bandwidth of 4 used to create Figures A and a black horizontal line identifies this bandwidth. The shading corresponds to the significance and direction of the slope (first derivative) of the WRR by age: red is significantly decreasing, purple is possibly zero, blue is significantly increasing, and light grey represents areas where there is insufficient data to generate a smoothed curve. The grid lines correspond to 1 year of age intervals. C SiZer plot of the second derivative of the WRR by age, where the shading corresponds to that described for Figure 1B. (DOC) [file pone.0042328.s001.doc]
